# Design and National Dissemination of the StrongWomen Community Strength Training Program

**Published:** 2007-12-15

**Authors:** Rebecca A Seguin, Christina D Economos, Miriam E Nelson, Raymond Hyatt, Ruth Palombo, Peter NT Reed

**Affiliations:** John Hancock Center for Physical Activity and Nutrition, Friedman School of Nutrition Science and Policy, Tufts University; John Hancock Center for Physical Activity and Nutrition, Friedman School of Nutrition Science and Policy, and Jonathan M. Tisch College of Citizenship and Public Service, Tufts University, Boston, Massachusetts; John Hancock Center for Physical Activity and Nutrition, Friedman School of Nutrition Science and Policy, and Jonathan M. Tisch College of Citizenship and Public Service, Tufts University, Boston, Massachusetts; John Hancock Center for Physical Activity and Nutrition and Friedman School of Nutrition Science and Policy, Tufts University, Boston, Massachusetts; Friedman School of Nutrition Science and Policy and Department of Public Health and Family Medicine, School of Medicine, Tufts University, Boston, Massachusetts; John Hancock Center for Physical Activity and Nutrition, Tufts University, Boston, Massachusetts

## Abstract

**Background:**

Physical activity is essential for maintaining health and function with age, especially among women. Strength training exercises combat weakness and frailty and mitigate the development of chronic disease. Community-based programs offer accessible opportunities for strength training.

**Program Design:**

The StrongWomen Program is an evidence-informed, community-based strength training program developed and disseminated to enable women aged 40 or older to maintain their strength, function, and independence. The StrongWomen Workshop and StrongWomen Tool Kit are the training and implementation tools for the StrongWomen Program. Program leaders are trained at the StrongWomen Workshop. They receive the StrongWomen Tool Kit and subsequent support to implement the program in their communities.

**Dissemination:**

Program dissemination began in May 2003 with a three-part approach: recruiting leaders and forming key partnerships, soliciting participant interest and supporting implementation, and promoting growth and sustainability.

**Assessment:**

We conducted site visits during the first year to assess curriculum adherence. We conducted a telephone survey to collect data on program leaders, participants, locations, and logistics. We used a database to track workshop locations and program leaders. As of July 2006, 881 leaders in 43 states were trained; leaders from 35 states had implemented programs.

**Conclusion:**

Evidence-informed strength training programs can be successful when dissemination occurs at the community level using trained leaders. This research demonstrates that hands-on training, a written manual, partnerships with key organizations, and leader support contributed to the successful dissemination of the StrongWomen Program. Results presented provide a model that may aid the dissemination of other community-based exercise programs.

## Background

### Aging and the value of strength training 

Physical inactivity and poor nutrition are leading contributors to chronic disease and premature death throughout the United States and abroad (1-3). As the average lifespan of Americans increases, older adults are becoming vulnerable to the effects of chronic disease, weakness, and functional decline. During aging, people often lose strength, muscle mass, and bone mass and decrease their levels of physical activity and dietary quality (4-6). The age-related loss of muscle and bone mass and their effects are more pronounced in women because women naturally have less muscle and bone mass than men and because the loss of lean tissue is accelerated during menopause (7-9). That loss of muscle mass may compromise a woman's ability and confidence to participate in regular exercise and to perform common daily activities, such as household chores (10-12).

Research has shown that many age-related physiologic declines are not inevitable. Laboratory and home-based studies have demonstrated that strength training — also referred to as progressive resistance training or weight lifting — confers numerous health benefits, particularly for women as they age. Strength training is an activity in which muscles move dynamically against weight (or other resistance) with small but consistent increases in the amount of weight being lifted over time. Done regularly, these exercises build bone and muscle and help to preserve strength, independence, and vitality (13-16). For instance, postmenopausal women aged 50 to 70 years increased bone and muscle mass, as well as strength, during 1 year of progressive strength training exercises while their age-matched counterparts, who did not strength train, experienced declines in these measures (17). In addition to reducing the risk of osteoporosis, strength training reduces risk for falls, lessens morbidity from diabetes and osteoarthritis in older adults, reduces depression, and improves sleep and self-confidence, according to randomized, controlled trials (13,16-22).

Despite compelling scientific research and recommendations from the government and the American College of Sports Medicine (ACSM), only 17% of adult women and approximately 12% of all adults aged 65 or older participate in strength training exercises (23-25). One objective of the U.S. Department of Health and Human Service's *Healthy People 2010* guidelines is to increase to 30% the proportion of adults who perform physical activities that enhance and maintain muscular strength and endurance (25).

### Community-based programming and community leaders 

Exercise programs can be executed in a variety of settings. People may choose to exercise at home, with a group at their faith-based organization, or at a sport and fitness facility. Home-based programs, for instance, involve an individual acquiring materials — including instructions and illustrations as well as background and motivational information — and then following the program at home. One example is the widely disseminated *Exercise: A Guide from the National Institute on Aging* (4). Home-based programs offer convenience and affordability but little opportunity for feedback or socializing. Other common venues for exercise are fitness clubs, where individuals purchase memberships that provide access to a range of equipment, instructors, and classes. Although this setting allows for feedback and social opportunities that are unavailable in home-based programs, it may present barriers such as cost, accessibility (i.e., location and transportation issues), and individuals' lack of confidence in using equipment or participating in classes.

Community-based exercise programs are similar to programs operated in fitness clubs in that they bring groups of participants together to exercise. In contrast, community-based exercise classes are held in public venues, such as local community or recreational centers, churches, county 4-H buildings, or public housing facilities; also, "membership" is simply being a member of that local community. Community-based programs often have a host organization that supports programs by providing equipment and generating publicity. Community-based exercise programs offer many advantages: they are typically more accessible, less expensive, and less intimidating than programs in fitness clubs, and they provide opportunities for feedback and social and peer support, which have a positive impact on long-term behavior change (26-35). Community-based programs have also been shown to increase knowledge and awareness of health-related behaviors (e.g., making healthy food choices) and to promote and support long-term behavior change (26-28). Because of these advantages, community-based exercise programs may be more feasible and sustainable than home-based programs or those requiring membership (29,30).

The StrongWomen Program is a community-based exercise program that focuses on increasing women's access to regular strength training opportunities and increasing knowledge about the importance of regular strength training (35-37). Community leaders assist in executing community-based programs in the following areas: administrative tasks (e.g., registration), program promotion (e.g., fliers, informational meetings), class organization, scheduling, set up, conducting the classes, and responding to program participants' questions, needs, and feedback. The formal title for a community leader who has been trained to implement the StrongWomen Program is StrongWomen Program Leader, hereafter referred to as program leader.

### Research, demographics, and the social environment 

Several factors converged in the 1990s to create a fertile environment for the dissemination of a community-based strength training program targeted to women. During this period, research was published that demonstrated the importance of lifting weights as age increases, particularly for women (13,16-19). The publication of the *Strong Women* books and similar publications translated much of the research into practical strategies for individual use (38-40). In addition, several other communication and media elements — ranging from television and radio to print and online publications — supported the message of the importance of strength training for women.

Concurrently, the absolute numbers of middle-aged and older women (aged 40 or older) was growing, increasing the number of potential program participants. From 1990 to 2000, the number of women aged 40 or older grew by 23.3%, compared with a 13.2% growth in the total population (41,42). Women were also increasingly engaged in their own health, becoming more educated about their options for maintaining good health as they approached midlife and becoming more empowered to engage actively in making healthful decisions (43-45).

An increased awareness and promotion of exercise at the local, state, and national levels fueled the interest in making healthier choices. In particular, the ACSM and the Centers for Disease Control and Prevention were publishing clear, discernible messages about the importance of physical activity in general and strength training in particular (14,46). Chapter 22 of the *Healthy People 2010* report presented data on strength training practices in the late 1990s and goals for 2010, including goals for the proportion of older adults to participate in strength training exercises (25,35,36).

This environment prompted the development of the StrongWomen Program. The goal of the program was to translate the strength training research into a practical application that program leaders could implement in their communities for a broad audience of women. With the growing interest and demand from the target population of women and the support of recent research, the timing was optimal for women to gather and work toward the goals of improved health and wellness. The StrongWomen Program was designed to meet these goals and to provide the additional benefit of a supportive social community of "strong women" program participants and leaders.

**Figure 1:**
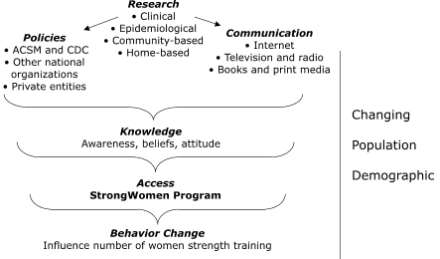
Contextual concept for development and dissemination of the StrongWomen Program, a community-based strength training program targeted to women aged 40 or older. ACSM indicates American College of Sports Medicine; CDC, Centers for Disease Control and Prevention.

The combination of a strong and growing research base, demographic changes in the target population, and the recognition that social support is an important element of participation in exercise programs made the development and dissemination of the StrongWomen Program timely (Figure 1).

## Program Design

The mission of the StrongWomen Program was to increase the health and vitality of middle-aged and older women across the country. To achieve this mission, the principal objective was to disseminate an easily sustainable, evidence-informed, community-based strength training program targeted to middle-aged and older women.

### Overcoming barriers to program implementation  

Barriers to implementing safe and effective exercise programs, particularly for older individuals, are common. They include program fees, physical accessibility, scheduling, equipment purchase, and identifying qualified leaders. One goal of the StrongWomen Program was to review existing research and publications to address potential barriers proactively during the development phase and, therefore, create a curriculum, training, and support system for leaders that would address and minimize potential obstacles to implementation and sustainability. A discussion of barriers and how to overcome them follows.

**Evidence-informed programming**

Many academic institutions contribute to the growing body of literature about the benefits of exercise for older adults. Unfortunately, little of this research reaches the public, and when it does, it is rarely translated into practical and accessible exercise programs. We based the StrongWomen Program on scientific research and public health recommendations that advise older adults to perform strength training exercises at least twice weekly; this foundation provides the essential element of credibility for the program (17,23-25,46-52).

**Community-based programming**

We designed the StrongWomen Program as a community-based program to be implemented in nonprofit community settings and organizations. We aimed to make the program as affordable, accessible, and approachable as possible and to use the social support of program participants and leaders to promote sustained behavior change. We encourage program leaders and their organizations to facilitate communication and networking among program participants.

**Educating health care providers**

Another barrier to exercise programs for older adults is the lack of knowledge among physicians and other health care providers about evidence-informed exercise programs that are available to their patients. The StrongWomen Program Tool Kit (described below) includes an easy-to-read information sheet that participants can give to their health care provider, along with a packet of peer-reviewed research articles detailing the scientific basis for the program. We distribute contact information for our staff and each site's leader to foster communication between health care providers and program administration and leaders (4-6,10-13,16-22).

### Experience of the StrongWomen Program participants 

StrongWomen Program classes last approximately 1 hour and consist of 5 minutes of warm up (e.g., walking, marching in place), 40 minutes of strength training, 5 minutes of balance training, and 5 minutes of cool down (i.e., stretching and flexibility exercises). The StrongWomen Program is a 12-week session with two 1-hour classes per week on nonconsecutive days. Generally, eight to 15 participants per class participate in the 12-week session as a group. Most program leaders operate subsequent sessions as a maintenance program for a group that has completed a 12-week session while initiating separate sessions for new groups. We encourage participants to perform the strength training exercises on their own on a third nonconsecutive day of the week. To assist them, leaders distribute copies of the exercise descriptions and illustrations as well as a list of local resources for other physical activity opportunities (e.g., walking clubs). Program leaders may choose to make minor adjustments to the recommended program. For example, they may schedule a 10-week class instead of a 12-week class.

The greatest variation in program logistics is in participant cost and equipment. The out-of-pocket cost to participants ranges from none (when no class fee is charged and equipment is provided by the program) to $120 for 12 weeks of classes ($5 per class twice per week), plus the need to bring their own equipment. A typical fee for a session in which all equipment is provided by the program is $48 to $96 for 12 weeks of classes (or $2–$4 per class). Ultimately, the program leader or the organization implementing the program determines the fees and how the equipment is acquired and paid for. The equipment per participant includes at least two sets of dumbbells (i.e., a 5-lb and an 8-lb pair), an adjustable ankle weight (10–20 lb per cuff), and an exercise mat or towel for floor exercises.

When participants must purchase their own equipment, it costs approximately $50 to $80 ($10–$15 for dumbbells, $30–$50 for a 20-lb ankle weight, and $10–$15 for an exercise mat). This estimate is for new equipment and includes shipping and handling fees. Obtaining used equipment and avoiding shipping and handling fees reduce costs substantially.

When program leaders provide the equipment, the cost varies but is typically less per participant than when the participants purchase their own equipment because weights and mats can be purchased at bulk discounts up to 50%. For example, the equipment cost for 10 participants ranges from $25 to $40 per participant (and less if used equipment can be obtained). The meeting space and other items that a program leader must provide to participants include an adequately sized, well-lit room; a parking area; sturdy chairs; and bathroom facilities.

### The StrongWomen Program curriculum: workshop and tool kit

The foundation of the StrongWomen Program is the written manual (the tool kit) and the hands-on training for program leaders (the workshop). Collectively, the workshop and tool kit form the curriculum for the program. Neither is a stand-alone entity; each potential leader must attend the workshop to receive the tool kit and subsequently implement the program.

**The StrongWomen Workshop**

During the full-day workshop (8 hours, including a working lunch), program leaders participate in a series of seminars and hands-on sessions based on the content of the tool kit. During the hands-on sessions, they learn how to instruct participants on the strength training and flexibility exercises. The tool kit describes and illustrates all exercises, and participants model, review, and practice them several times throughout the workshop.

The workshop also introduces program leaders to the two types of assessment and evaluation tools that can be used to measure participants' progress and satisfaction with the program. One tool is a questionnaire designed to help program leaders receive detailed subjective feedback from participants about a range of topics related to their program. The second is an objective measure of change in physical parameters that relate to program participation, including muscular strength, endurance, agility, flexibility, and balance. This second tool is excerpted with permission from the *Senior Fitness Test* (53); it provides norms for each physical assessment for women aged 60 or older.

Proactively minimizing barriers to participation is a priority for increasing access to the program, and the workshop, therefore, includes a 30-minute brainstorming session to address issues related to fees and costs. We strongly encourage program leaders to assist and facilitate participation by any individual who is interested in joining the class, regardless of income. A few of the strategies discussed during the brainstorming session have included soliciting donations (e.g., equipment, money, space, participant incentives such as T-shirts and water bottles) and negotiating discounts from local vendors and organizations. We transcribe notes from the discussion as well as other questions and answers posed during the workshop and distribute them to program leaders at the end of the day. Workshop attendance at Tufts University is $300 per attendee and includes the StrongWomen Program tool kit as well as breakfast and lunch. Cost of attendance at off-site workshops varies, depending on sponsorship and resources, but it never exceeds the $300 fee.

**The StrongWomen Tool Kit**

The StrongWomen Tool Kit (54) is a 200-page binder that includes the information and supporting materials that a program leader needs to implement and maintain the StrongWomen Program. In addition to the main content, the tool kit includes several sets of separately collated handouts that are intended for use with participants, their health care providers, and the news media. These handouts include nutrition fact sheets (to give to participants), a packet of peer-reviewed journal articles outlining the benefits of strength training (to give to health care providers), and a sample press release and program summary sheet (to give to the news media). In addition, we provide a physician consent-to-exercise document; we strongly suggest that leaders collect physician consent forms for all participants, but we do not require that they do so.  Table 1 presents the tool kit table of contents (54).

## Dissemination

Dissemination began in May 2003 in three parts: recruiting leaders and forming key partnerships, soliciting participant interest and supporting program implementation, and promoting growth and sustainability.

### Part 1: Recruiting leaders and forming key partnerships 

The first group of program leaders were members of organizations that have since become key partners with the StrongWomen Program: hospitals, nonprofit outpatient wellness centers, and the National Extension Association of Family and Consumer Sciences branch of the Cooperative State Research, Education, and Extension Services (hereafter referred to as the Extension Service), which is under the direction of the U.S. Department of Agriculture. Individuals from these organizations had seen the *Strong Women* books and were interested in operating programs within their own organizations on the basis of the research and practical applications presented in the books. They contacted Tufts University with their interest, and the StrongWomen Program began shortly thereafter.

Hospitals and nonprofit outpatient wellness centers — in particular, women's wellness centers — are important for the StrongWomen Program because they are often at the core of women's health services within a community. In addition to offering standard medical care, such as family practice, gynecology, and endocrinology, women's wellness centers may offer community programming such as outpatient exercise and nutrition classes. Hospitals are also important for the StrongWomen Program because they provide a large audience of potential program participants and they usually have the space and resources necessary for implementing and maintaining the program. Because the StrongWomen Program is in-house at a hospital or wellness center, physicians perceive the program as a safe and viable option for their patients.

Extension Service educators, who are in every county in every state, offer research-based health information and programs to their communities. Collaboration with the StrongWomen Program helps Extension Service agents to bring knowledge, awareness, education, and research-based programming to community members and to increase the Extension Service's reach in underserved and rural locations (a focus area for the Extension Service).

Since the inception of the StrongWomen Program, collaboration with clinics and the Extension Service has been vital to disseminating the program. Knowledge of the program has spread within the networks of these organizations by internal newsletters, bulletin boards, word-of-mouth, and formal presentations at professional meetings. At the national conference of the Extension Service in October 2004, we trained 150 program leaders at a StrongWomen Program Workshop. (Most workshops have 15–40 attendees.) The broad geographic range represented by program leaders at this workshop expanded the dissemination of the StrongWomen Program and supported grassroots awareness.

Prospective program leaders also learn about the StrongWomen Program through the *Strong Women* book series and its related Web site (www.strongwomen.com) and the StrongWomen Program Web site (go.tufts.edu/strongwomen), which can be accessed directly or linked through www.strongwomen.com (38,39). Through www.strongwomen.com, any individual can sign up to receive the free monthly electronic newsletter, which includes the following: a research update, such as new study findings and take-away messages; upcoming public talks, forums, summits, and events; upcoming StrongWomen Workshops; reader questions and our answers; reader success stories; and a recipe of the month. This newsletter has approximately 26,000 subscribers. The StrongWomen Program Web site contains details about the program, the workshop agenda, and upcoming workshop dates and locations. Individuals can contact the program manager through this Web site to request to be added to an e-mail list for upcoming workshop announcements, which are sent regularly throughout the year.

Some program leaders learn about the program and training through the books, Web sites, or some other way, and, therefore, attend the workshop on their own initiative; many program leaders are sent by their employer or an organization. We now require program leaders to be strength training actively at the time they attend the workshop; until the 2004 telephone survey (detailed later in this article), this requirement was only a strong recommendation. In addition, program leaders must implement the StrongWomen Program only in nonprofit organizations, such as senior centers, hospital outpatient centers, Extension Service locations, assisted living facilities, or faith-based organizations. A simple preregistration worksheet assesses the qualifications for nonprofit status.

The StrongWomen Program highly recommends, but does not require, that program leaders have at least two of the following: an educational background in a field such as health services, nutrition, exercise physiology, physical therapy, or public health; some experience in providing exercise instruction; and certification by a reputable health and fitness organization, such as the National Strength and Conditioning Association, the ACSM, or the American Council on Exercise. As of July 2006, program leaders have ranged in age from 21 to 83 years, with a mean age of 50 years, and have had a diverse range of professional backgrounds (Table 2).

### Part 2: Soliciting participant interest and supporting program implementation

To help new program leaders implement the StrongWomen Program in their communities, we encourage them to follow several steps: 1) read the entire tool kit within 1 week of the workshop; 2) find an organization to host the program and a space to operate the program (if they do not already have ties to a hosting organization); 3) determine the pricing structure, equipment, and schedule for their StrongWomen Program, working with their hosting organization; 4) plan and publicize an informational meeting about the program using the publicity materials provided in the tool kit or other means; and 5) operate their first StrongWomen Program within 3 to 6 months of attending the workshop. New and experienced program leaders can receive support for implementing the StrongWomen Program by e-mail or telephone from the program manager or by networking with other program leaders, many of whom are listed on the StrongWomen Program Web site.

Virtually anyone can be a participant in the StrongWomen Program. Program leaders recruit participants through placing advertisements in local newspapers, posting fliers throughout the community, or making announcements through available newsletters and bulletins. The research that provides the scientific basis for the program was conducted with women aged 40 to 91 years; we developed the exercise programs with this audience in mind. However, women may begin to lose muscle and bone mass at an earlier age, and we encourage program leaders to allow all women, regardless of age, to join the classes. As of July 2006, the age range of participants was 21 to 94 years with a mean of 63 years. Strength training is important for men as well; although we encourage program leaders to include men who wish to join, preliminary data show that most participants are women.

### Part 3: Promoting growth and sustainability 

A variety of mechanisms are in place for long-term maintenance of the StrongWomen Program. Two maintenance objectives are to educate current and potential program leaders using the most up-to-date evidence-informed programming possible and to continue to assist them in implementing and sustaining their programs.

The program manager spends approximately 15 to 20 hours per week answering 200 to 300 e-mails and 40 to 60 telephone calls from program leaders. The program manager responds to questions from prospective program leaders who are considering attending a workshop and from current program leaders to support implementation of existing programs. This support includes assisting with space, equipment, and resource issues; helping with incentive and reward programs for participants; working with volunteer assistants to program leaders on class set-up and other issues; and helping program leaders identify modifications for exercises to improve accessibility for some participants. The program manager also publicizes continuing education events and curriculum updates among program leaders through the e-mail list and the StrongWomen Program Web site.

The StrongWomen Ambassador training program is another component of program growth and sustainability. Seven ambassadors conduct workshops in Alaska, Arkansas, Colorado, Kansas, Oregon, and Pennsylvania. These individuals participate in a more extensive training process than the workshop provides, and they are then qualified to hold workshops within their own states to train new program leaders. Ambassadors are also important for program sustainability by serving as local resources for program leaders. To become an ambassador, a program leader must have been actively operating programs in his or her state for at least 6 months. Then he or she must attend a second program leader workshop, which is identical to the first. At this second workshop, we give the teaching materials to the potential ambassadors and instruct them to observe the *teaching process* instead of the workshop content. After the second workshop, we require prospective ambassadors to plan, execute, and follow up on their own workshop attended by members of the public and their hosting organization. In addition to allowing the candidate to demonstrate a mastery of the entire curriculum, this workshop provides an opportunity for the candidate to demonstrate support from his or her sponsoring organization; both demonstrations are requirements for becoming an ambassador. The program manager determines the guidelines and protocols in collaboration with each potential ambassador and the hosting organization (because logistics may vary site by site) and attends the workshop to oversee its complete execution.

The aims for long-term sustainability of the StrongWomen Program are to focus efforts on creating supplementary curriculum materials, such as additional evidence-informed exercise programs that participants will require as their strength and fitness increase; on facilitating the leadership and training of additional ambassadors, who are critical to maintaining the reach and momentum of the program's growth; and on creating advanced workshops and educational opportunities for program leaders that will enable them to broaden the scope and capacity of their leadership as agents of positive change in their communities.

## Assessment

### Adherence to the curriculum — site visits 

The program manager conducted site visits at six active StrongWomen Program classes in Kansas, Oregon, and Massachusetts during the first year of dissemination. The primary component of the site visit was observation. During the observation, the program manager observed one or more complete exercise sessions at each site and graded each of the following on a 5-point scale (1 = unacceptable, 2 = needs improvement, 3 = fair, 4 = good, 5 = excellent):

- Adequate space for participants and equipment (i.e., movement through range of motion)
- Equipment safety (e.g., sturdy chairs, appropriate dumbbells, nothing makeshift)
- Location safety and accessibility (e.g., availability of parking, adequate lighting, dry floors)
- Execution of exercise program (i.e., proper use of equipment, speed, demonstration and feedback on exercise form, rest periods, and verbal prompting and encouragement)

Following the observation, the program manager conducted interviews with participants as a group and with leaders individually. Interviews were related to program logistics (e.g., scheduling, class length), level of participant comfort with their leader, opinions on enjoyment of the program, perceived benefits, and suggestions for changes.

The results revealed adherence to the curriculum in terms of space, equipment, location, and exercise program recommendations outlined in the tool kit. Adherence was determined by an average score of at least 4 in all categories. In Kansas, two classes at one site scored an average of 4.25; in Oregon, three classes at one site scored an average of 4.5; and in Massachusetts, three classes at one site scored an average of 4.25.

Both program leaders and participants were satisfied with the program logistics and outcomes related to participation. The primary requests from program leaders were related to more guidance on fee structures and scheduling, which is now addressed in greater detail during the workshop. Participant concerns were related to scheduling and the desire for additional nutrition information. Scheduling concerns were subsequently addressed with leaders and expanded upon in the tool kit. Although the took kit already included a chapter on nutrition, a packet of fact sheets on nutrition (similar in content to the information presented to program leaders in the tool kit) is now available for program leaders to distribute to participants.

### Telephone survey 

By September 2004, 142 program leaders from 13 states had been trained; 139 were women. Of the 139 women, 31 (22%) were from urban areas, 55 (40%) were from suburban areas, and 53 (38%) were from rural areas. Of 130 program leaders with current contact information, 103 participated in a brief telephone survey (response rate, 79%). Of the 103 respondents, 72 (70%) had implemented at least one StrongWomen Program, with a mean class size of 11. The mean time between attending the workshop and starting the first program was 12 weeks (SD, 13 weeks). On the basis of logistic regression analysis that we performed for a previous study (55), we found that program leaders who had strength trained themselves before attending the workshop were more likely to have implemented the StrongWomen Program. We also found that program leaders sent by their employer or an organization to attend the workshop were more likely to have implemented the program than were program leaders who attended the workshop on their own initiative (55). We have conducted additional follow-up surveys with program leaders and participants; findings from these data are forthcoming.

### Program leader and participant databases 

Although we recommend that program leaders return to their communities to implement the StrongWomen Program within 3 to 6 months of the workshop, only some do. Because the program's mission is to increase access to and participation in strength training programs by middle-aged and older women, we track the number of program leaders, their locations across the country, and the number of StrongWomen Programs they implement. We established two databases for this purpose, one for program leaders and one for participants.

We use the program leader database to collect and maintain contact information and an accurate count and geographic distribution of program leaders, whether they were trained at Tufts University or by an ambassador elsewhere in the country. We use these data to query program leaders about program implementation and participant compliance and to obtain their qualitative feedback on program and curriculum needs for the future. We use the participant database, which was populated by data collected by program leaders with participants' consent, to conduct research related to long-term adherence to the behavior of strength training. We have also used contact information from this database to obtain feedback from participants on the StrongWomen Program curriculum and leaders. We sent surveys to all participants and program leaders in our databases in July 2006. We present basic data from these surveys (e.g., mean age and age range of program leaders and participants) in this article; most of those data and their analysis (e.g., participant compliance) are forthcoming.

**Figure 2:**
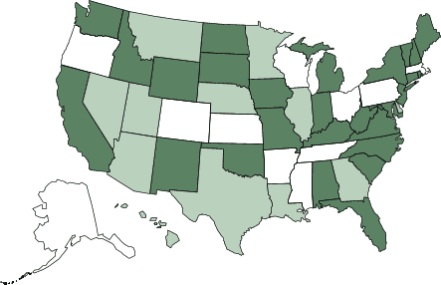
Active StrongWomen programs and workshop sites as of July 2006. Dark green indicates states with active StrongWomen Program classes (plus Ontario, Canada, not shown); white, states with active classes and at least one workshop site; light green, states with no StrongWomen Program classes or workshop sites.

As of July 2006, 39 workshops had been conducted for 881 program leaders from 43 states. Thirty-eight states have active StrongWomen Programs (Figure 2). On the basis of the 70% implementation rate and average class size of 11 participants reported in the telephone survey, we conservatively estimate that 6800 people had participated in StrongWomen Programs across the country by July 2006. However, because many program leaders conduct concurrent sessions (e.g., one program leader may have three different groups, each meeting twice per week for a total of 33 participants), the true number of participants is likely to be greater.

## Conclusion

Our objective to increase middle-aged and older women's access to community-based strength training programs was timely because of the growth of this population, current physical activity recommendations, and increased awareness of the benefits and importance of strength training for women as they age. The monthly StrongWomen newsletter, Web sites, and other correspondence (e.g., telephone, e-mail) offered low-maintenance vehicles for regular communication with both existing and potential program leaders as well as leaders from key organizations.

Dissemination relied heavily upon establishing solid partnerships within stable organizational networks (i.e., hospital-based wellness centers and the Extension Service). An evidence-informed program, a straightforward curriculum, and the flexibility to implement the program in a range of sites facilitated the successful implementation by program leaders. For participants, the credibility, accessibility, and affordability as well as the social and peer support inherent in the community-based structure of the program likely contributed to their participation.

The mission of public health is to prevent disease and promote health in the greater population. Among a myriad of important issues, widespread access to exercise opportunities is a public health priority, and community-based programs present a feasible strategy for addressing it. The national dissemination of this strength-training program targeted to middle-aged and older women provides a viable model and systematic method for increasing access to evidence-informed exercise programming in a range of community settings.

## Figures and Tables

**Table 1:** Table of Contents for Tool Kit, StrongWomen Community Strength Training Program, 2006

Caution	A note about implementing community exercise programs
Foreword	The inspiration and motivation to develop the program
Mission and Objectives	The mission statement and objectives for the program
Chapter 1	The benefits of strength training for women — the research behind the program
Chapter 2	Starting a program — leaders, sites, space, equipment, promotion, and scheduling
Chapter 3	Participant screening — contact information, medical history, screening tools, and consent
Chapter 4	StrongWomen Program — two strength training programs, general exercise safety
Chapter 5	Keeping track and participant assessments — contact and attendance sheets, exercise logs, evaluations, and assessment tests
Chapter 6	Leadership — leader styles, skills, professionalism, courtesy and respect, communication, and selecting peer leaders
Chapter 7	General physical activity — different modes, walking programs, community involvement
Chapter 8	Nutrition for optimal health
Chapter 9	Frequently asked questions
Chapter 10	Resources
Acknowledgments	Gratitude for individuals and organizations that supported program development
References	Research citations
Handouts	Research packet, tracking packet, nutrition fact sheets, informational/media packet

**Table 2:** Program Leaders (N = 881) by Occupation, StrongWomen Community Strength Training Program, 2006

Occupation	No. (%)
Extension agent	379 (43.0)
Fitness instructor or personal trainer	69 (7.8)
Physician or nurse	36 (4.1)
Physical therapist	16 (1.8)
Nutritionist or dietician	14 (1.6)
Other health care worker	52 (5.9)
Community educator or community organizer	51 (5.8)
Academic educator	18 (2.0)
Student	11 (1.2)
Self-employed	12 (1.4)
Other	96 (10.9)
Data field blank on registration form	127 (14.4)
