# Erratum, Vol. 5: Issue 1

**Published:** 2008-03-15

Because of an editing error in the article "Design and National Dissemination of the StrongWomen Community Strength Training Program," which appeared in the January 2008 issue, the author Peter N.T. Reed was incorrectly identified as having an MS degree. This designation should have been indicated as an MPH. The correction to the byline was made on December 18, 2007, and appears online at www.cdc.gov/pcd/issues/2008/jan/06_0165.htm. We regret any confusion or inconvenience this error may have caused.

